# BEAMSTER: Brain mEtAstases segMentation for STEreotactic Radiotherapy, A Retrospective MRI Dataset with Expert Segmentations

**DOI:** 10.1038/s41597-026-07777-0

**Published:** 2026-07-01

**Authors:** Michal Nohel, Stefan Reguli, Romana Kaplanova, Jana Jackaninova, Jiri Chmelik, Lukas Knybel

**Affiliations:** 1 Department of Deputy Director for Science and Research, University Hospital & Faculty of Medicine, Ostrava, 708 52 Czech Republic; 2 Department of Biomedical Engineering, Faculty of Electrical Engineering and Communication, Brno University of Technology, Brno, 616 00 Czech Republic; 3 Department of Neurosurgery, University Hospital & Faculty of Medicine, Ostrava, 708 52 Czech Republic; 4 Department of Oncology, University Hospital & Faculty of Medicine, Ostrava, 708 52 Czech Republic

## Abstract

We present a retrospective dataset of contrast-enhanced T1-weighted magnetic resonance imaging scans from 140 patients with brain metastases who underwent stereotactic radiotherapy. In total, 260 metastatic lesions were annotated by an experienced radiation oncologist using a radiotherapy planning system, and the resulting binary segmentation masks were converted into NIfTI format. All data were de-identified prior to release, and facial features were removed from imaging data using a defacing procedure to ensure patient privacy. The dataset represents a valuable resource for the development and validation of computer-aided detection and segmentation methods, including those based on machine learning. A particular strength is the inclusion of small metastatic lesions, which are clinically relevant yet challenging to detect and segment automatically. We anticipate that this collection will facilitate reproducible research and support the development of robust algorithms for clinical translation in radiotherapy planning.

## Background & Summary

Oncological diseases currently represent the second most common cause of death, after cardiovascular diseases. Brain metastases (BMs) occur in 20–40% of patients with a primary extracerebral malignancy^1^, making them the most frequent type of brain tumor. The highest risk of CNS metastasis is associated with lung carcinoma, melanoma, renal cell carcinoma, breast carcinoma, and colorectal carcinoma^2^. Approximately 40 to 50% of patients with BMs have a solitary brain lesion, while 50 to 60% develop multiple metastases^1^.

Brain metastases can be managed surgically, with radiation therapy, or with a combination of both. According to international guidelines (e.g., EANO-ESMO^3^ and ASTRO^4^), surgery is indicated primarily for patients with a large symptomatic lesion when resection can be performed without major neurological deterioration, or in cases where histological confirmation is required^3–5^. In most other scenarios, stereotactic radiosurgery (SRS) or stereotactic body radiotherapy (SBRT) represents the treatment of choice.

Magnetic resonance imaging (MRI) is the gold standard for diagnosing metastatic brain involvement^6^. In the vast majority of cases, brain metastases appear as well-delineated contrast-enhancing lesions on T1-weighted post-contrast images, which serve as the basis for radiation planning and further follow-up.

Within SBRT planning, the most time-consuming task for clinicians is to define both the target volume (metastasis itself) and the organs at risk, which must be spared from irradiation^7^. Precise segmentation of metastatic lesions is essential for selecting the appropriate therapeutic approach. Identifying and delineating small lesions is particularly labor-intensive and prone to error due to potential omissions^8^.

Changes in irradiated metastases can be assessed based on the volume of contrast-enhancing lesions on follow-up MRI scans (MRI volumetry)^9^. Reliable automatic segmentation could substantially reduce workload and potentially improve the accuracy and reproducibility of imaging assessments.

In recent years, several public datasets have become available to support research on brain metastases. Among the most widely used are the BraTS-METS 2023 dataset^10^, released as part of the Brain Tumor Segmentation (BraTS) Challenge, and the Large Open Access Dataset of Brain Metastasis 3D Segmentations^11^, both providing multi-parametric MRI data with expert annotations, typically focused on clinically visible metastases, with limited explicit stratification by lesion size. Other important resources include BrainMetShare^12,13^, the Yale Brain Metastases Longitudinal Dataset^14^, and the UCSF Brain Metastases Segmentation Resource^15^, which together offer various imaging data and annotations that support the development and validation of automated methods for detection, segmentation, and longitudinal analysis of brain metastases.

Accurate detection of very small brain metastases remains a major challenge in both clinical practice and the development of artificial intelligence tools. Small lesions are more prone to interobserver variability in manual delineation and are frequently underrepresented in clinical datasets, leading to bias in model training. Enriching the dataset with such cases increases the likelihood that a neural network can learn the subtle imaging features associated with sub-centimeter metastases, thereby improving sensitivity without compromising specificity. From a clinical perspective, early identification of these lesions is critical, as it can allow treatment in a stage where SRS remains feasible within safe dosevolume limits for normal brain tissue.

In this study, we introduce a retrospective dataset comprising 140 patients diagnosed with brain metastases, all of whom underwent radiotherapy treatment, with a total of 260 annotated metastatic lesions. A key characteristic of the dataset is a high proportion of small brain metastases, which are often underrepresented in existing open-access datasets and therefore provide a challenging setting for automated detection and segmentation methods. For each patient, metastatic lesions were manually segmented by an expert radiation oncologist, focusing specifically on the lesions that were selected for irradiation. The dataset includes imaging and segmentation data from routine clinical workflows, ensuring realistic anatomical and treatment variability. This dataset may serve as a valuable resource for the development and objective evaluation of computer-aided detection and diagnostic (CAD) systems, particularly those employing machine learning and artificial intelligence methods, which have seen rapid advances in recent years.

**Fig. 1:**
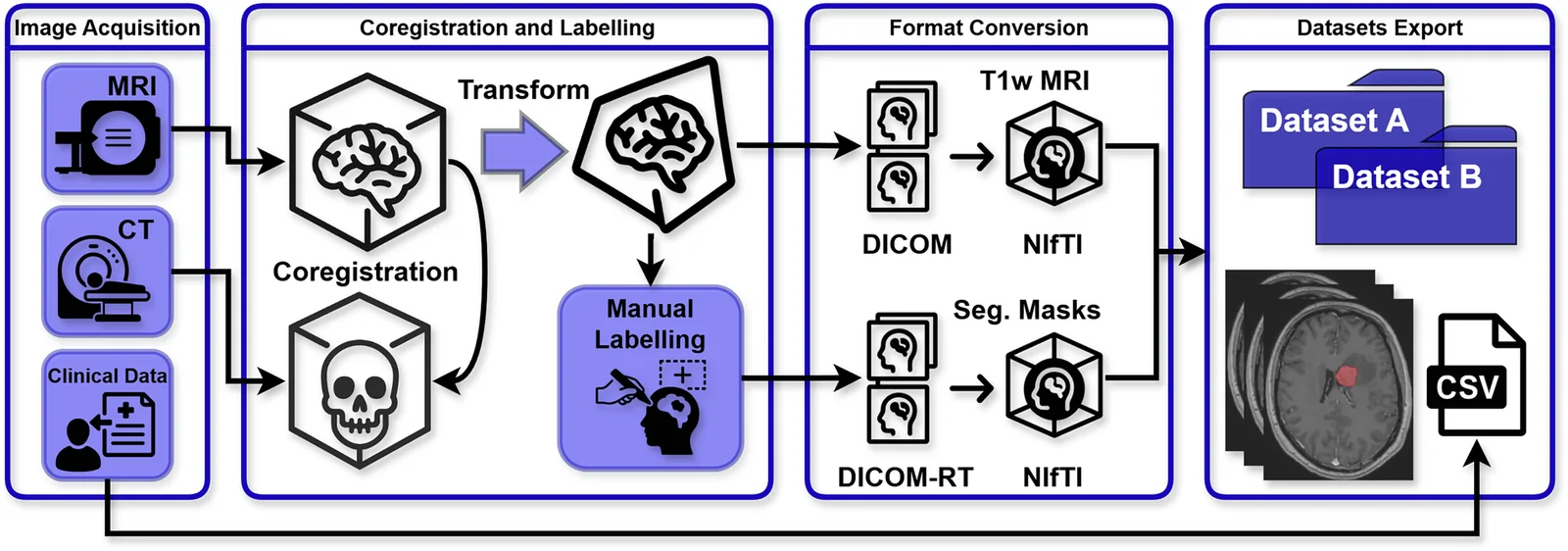
Graphical Abstract. Overview of the dataset creation pipeline and data structure. The process includes the acquisition of T1-weighted MRI scans and planning CT data, followed by rigid registration of MRI to CT. Brain metastases were manually delineated by a radiation oncologist (with 13 years of experience) within the radiotherapy planning system. The resulting contours were stored in the DICOM RT structure set and subsequently converted into binary NIfTI segmentation masks. In parallel, all imaging data were exported from DICOM into NIfTI format to ensure compatibility with standard neuroimaging pipelines. Relevant clinical metadata were collected and curated for each patient. The final dataset, therefore, consists of paired MRI scans and expert-derived segmentation masks, together with structured clinical data, all fully anonymized and prepared for public release. All images and segmentations were visualized and prepared using MITK software^22^ (version v2024.06).

A graphical abstract summarizing the data acquisition, processing pipeline, and dataset structure is provided in Fig. 1.

## Methods

### Patient selection

A retrospective analysis was conducted on consecutive patients who underwent brain MRI examinations at the Department of Oncology, University Hospital Ostrava, using a Magnetom Avanto 1.5 T, Magnetom Sola 1.5 T, or 3 T Magnetom Prisma scanner (all manufactured by Siemens Healthineers, Erlangen, Germany) between October 2019 and September 2024.

The dataset included only navigation MRI scans acquired for stereotactic radiotherapy planning with the CyberKnife system (Accuray Inc., Sunnyvale, CA, USA). The diagnosis of brain metastases had been established earlier using standard diagnostic imaging and the treatment strategy was determined by a multidisciplinary team.

The cohort included a heterogeneous distribution of primary malignancies. Non-small cell lung cancer (31/140, 22.1%) represented the most frequent histologically confirmed subtype, followed by melanoma (21/140, 15.0%), renal cell carcinoma (15/140, 10.7%), and breast cancer (13/140, 9.3%). Less common primary sites included gastrointestinal malignancies (11/140, 7.9%), bronchogenic carcinoma of unspecified subtype (10/140, 7.1%), and sporadic cases of colorectal, ovarian, and sigmoid colon carcinomas, as well as other specified but not further subclassified malignancies. In 17 cases (12.1%), the primary tumor remained unverified, reflecting metastatic disease with unknown primary origin.

It should be noted that the level of specificity of primary tumour classification was not uniform across the cohort. In earlier cases, the primary tumour was recorded at the level of anatomical site (e.g., lung or gastrointestinal tract), whereas in more recent cases a histologically confirmed diagnosis was available. This variation reflects changes in clinical documentation practices over time in this retrospective dataset.

The dataset was collected in two phases. Dataset A comprised cases treated with stereotactic radiotherapy in 1, 3, or 5 fractions between October 2019 and April 2022. All patients were routinely followed by the local neurosurgical team. This cohort consisted of 113 patients with a total of 216 brain metastases (mean volume 6.7 cc, SD = 10.5 cc).

Dataset B included cases treated with SRS between July 2021 and September 2024. This cohort consisted of 27 patients with 44 metastases (mean volume 0.5 cc, SD = 0.6 cc) and was specifically designed to enrich the dataset with very small lesions to improve the performance of a deep learning-based detection model under development.

Where possible, cases in Dataset B were selected to represent first-line local treatment of the target lesion, without prior radiotherapy to the same intracranial site. However, prior neurosurgical resection was present in a subset of patients (8 patients in Dataset A and 2 patients in Dataset B). In addition, the dataset includes both primary and re-irradiation cases. Treatment status (“primary” vs. “relapse”) refers strictly to the treatment history of the irradiated lesion: “relapse” indicates that the lesion had been previously locally treated, either by surgery or radiotherapy. Prior neurosurgical resection may or may not coincide with relapse status, as surgery could have been performed at a different intracranial site or for a different lesion.

Eligibility for SRS required compliance with established dose-volume constraints for normal brain tissue, including the planning target volume (PTV). Specifically, the volume of brain tissue receiving 12 Gy (V12 Gy) had to remain below 10 cc^4,16,17^, with a more stringent institutional constraint of V12 Gy < 7.85 cc.

Of all included patients, 45% were female, the mean age was 62.5 years (SD = 12.5). Histological verification was not performed in 12.1% (17/140) of the cases.

### Image acquisition

All MRI data were acquired as part of routine radiotherapy planning procedures (navigation imaging), using three different MRI scanners at the University Hospital Ostrava. Most scans were obtained on a Magnetom Avanto 1.5 T system, with a smaller number of more recent cases (n = 4) acquired on a Magnetom Sola 1.5 T. In occasional cases where standard scanners were unavailable for technical reasons, patients were imaged on a Magnetom Prisma 3 T system (n = 10).

Regardless of the scanner used, all patients underwent a standardized contrast-enhanced T1-weighted 3D MPRAGE (3D magnetization-prepared rapid gradient-echo) sequence, specifically the TFL3D (Turbo Fast Low-Angle) variant. Scanner-specific acquisition parameters are summarized in Table 1.

**Table 1:** MRI acquisition parameters for individual scanners used in the dataset.

Scanner	TR (ms)	TE (ms)	TI (ms)	Flip angle	Voxel size (mm)
Magnetom Avanto 1.5 T	1900	3.37	1100	15°	1.0 × 1.0 × 1.0
Magnetom Sola 1.5 T	2200	3.12	955	8°	0.9 × 0.9 × 0.9
Magnetom Prisma 3 T	1760	2.43	900	8°	0.9 × 0.9 × 0.9

All scans correspond to post-contrast T1-weighted 3D MPRAGE acquisitions. TR - repetition time, TE - echo time, TI - inversion time.

All MRI scanners used in this study, including the 3 T system, were calibrated and verified to ensure minimal geometric distortion related to magnetic field inhomogeneities, in accordance with institutional quality assurance protocols.

In addition to MRI, each patient also underwent non-contrast computed tomography (CT) as part of the radiotherapy planning process. CT scans were acquired using a Somatom Sensation Open scanner (up to 12/2022) and Somatom X.cite (from 1/2023), both manufactured by Siemens Healthineers, Erlangen, Germany, following a standardized radiotherapy simulation protocol in the supine position with the head immobilized in a 3-point thermoplastic mask to ensure reproducible positioning for radiotherapy planning. Although acquisition parameters were standardized, minor variations in voxel spacing may occur due to field-of-view selection and reconstruction settings. Subject-specific voxel spacing information is provided in the publicly available metadata file (image_voxel_parameters). CT voxel spacing varied across patients, with in-plane resolution ranging from 0.43 × 0.43 mm to 1.37 × 1.37 mm and slice thickness ranging from 0.625 mm to 1.25 mm; however, the majority of scans had a voxel spacing of approximately 0.5 × 0.5 × 1.0 mm.

### Image segmentation

All lesion segmentations were performed using MultiPlan 5.2 software (Accuray Inc., Sunnyvale, CA, USA). The segmentations were created by a board-certified radiation oncologist with 13 years of clinical experience based on registered MRI and CT scans.

The MRI datasets were rigidly registered to the planning CT, which served as the anatomical reference for radiotherapy planning and dose calculation. Registration was performed within the MultiPlan 5.2 software using a semi-automatic algorithm based on anatomical landmarks defined by the physician in both image sets. Consequently, all final segmentations and exported image volumes were aligned to the CT coordinate space and spatial resolution.

Segmentations were performed according to international radiation therapy guidelines, specifically following the volume definitions outlined in ICRU Report 50 Prescribing, Recording, and Reporting Photon Beam Therapy^18^. For each case, a Gross Tumor Volume (GTV) and a Planning Target Volume (PTV) were defined. In selected cases where a zero-margin approach was employed, the GTV was identical to the PTV. All contours were stored in the DICOM-RT Structure Set (RTSS) format and associated with the corresponding planning CT datasets.

For this study, only segmentations corresponding to brain metastases were extracted. The selected structures (GTV or PTV, depending on availability) were subsequently converted to the NIfTI file format for further processing and analysis.

### Imaging data anonymisation

All imaging data were de-identified in accordance with the General Data Protection Regulation (GDPR; Regulation (EU) 2016/679) and applicable Czech national legislation on personal data protection. Patient identifiers were removed during the initial export from the MultiPlan planning system in DICOM format, according to institutional and legal privacy standards. The DICOM files were subsequently converted to the NIfTI format to ensure compatibility with common neuroimaging analysis pipelines.

To further protect patient identity while preserving intracranial structures, an additional anonymization step was performed using the Analysis of Functional NeuroImages (AFNI) software package^19^. Specifically, the deface mode was applied, which removes facial features and ears while leaving all relevant brain structures intact. This two-step anonymization process ensures that the shared data are de-identified and suitable for public release.

### Dataset limitations

Although the dataset provides high-quality MRI scans with expert annotations, several limitations should be acknowledged.

First, this is a retrospective, single-institution study, which may introduce selection bias and limit generalizability. However, our institution is a high-volume centre for patients with brain metastases, and MRI examinations were selected from the institutional registry independent of tumour type, treatment strategy, image quality, or clinical outcome. MRI acquisition followed a standardized clinical protocol for the detection and assessment of brain metastases using standard-of-care sequences. Therefore, while selection bias cannot be fully excluded, the risk of systematic bias is likely reduced.

Second, only contrast-enhanced T1-weighted post-contrast MRI scans are included, which may limit applicability in multiparametric imaging studies.

Third, follow-up information in the dataset is limited to vital status and overall survival derived from date of death. Detailed systemic oncological treatment data were not systematically collected as part of the dataset design. However, additional clinically relevant local treatment variables were included, including prior neurosurgical resection and whether stereotactic radiotherapy to the indexed lesion represented primary treatment, postoperative treatment, or re-irradiation.

Finally, although annotations were performed by an experienced radiation oncologist, they represent single-observer segmentations and therefore do not capture inter-observer variability. In addition, images were acquired on multiple MRI scanners with different field strengths (1.5 T and 3 T), which introduces variability in image intensity and contrast despite standardized acquisition protocols. No post-processing harmonization or intensity normalization was applied, preserving the native heterogeneity of the dataset and enabling application-specific preprocessing strategies.

### Ethical approval

The study was conducted in accordance with the principles of the Declaration of Helsinki and was approved by the Institutional Review Board (Ethics Committee) of Ostrava University Hospital under the application registration number 352/2024. Data were collected retrospectively from routine clinical practice. Prior to inclusion, all patient identifiers and facial features were removed through anonymization procedures.

In accordance with applicable data protection regulations, a waiver of informed consent was granted by the Institutional Ethics Committee.

## Data Records

The dataset is available at the FigShare repository (https://doi.org/10.6084/m9.figshare.29365844)^20^ and is released under the CC-BY 4.0 license.

The dataset is organized into a single root folder named Dataset_BEAMSTER, which contains three subfolders: Dataset_A, Dataset_B, and Spreadsheets.

The Dataset_A and Dataset_B folders contain paired imaging data and corresponding segmentation masks in NIfTI format (.nii.gz). Each MRI volume follows the naming convention Dataset_X_YYY.nii.gz, and the corresponding segmentation mask is named Dataset_X_YYY_segm.nii.gz, where X denotes the dataset split (A or B) and YYY represents the subject identifier. MRI volumes and corresponding segmentation masks are stored in NIfTI format and provided in the planning CT coordinate space.

The Spreadsheets folder contains two files: Table_clinical_data.csv/.xlsx, which includes clinical variables for each subject, and image_voxel_parameters.csv/.xlsx, which provides voxel-level parameters associated with the MRI data. The spreadsheets are provided in both .csv and .xlsx formats for compatibility with common analysis pipelines.

In addition to imaging data, the dataset includes clinical information for each subject. Available attributes comprise patient age, sex, MRI scanner type, prescribed radiation dose, number of fractions, isodose level, prior neurosurgical intervention, treatment status of the irradiated lesion (primary treatment versus relapse/re-irradiation), and overall survival time (defined as the interval in days between the reference imaging examination and death). In cases where the origin of the metastasis was verified, the underlying primary diagnosis was also reported.

**Fig. 2:**
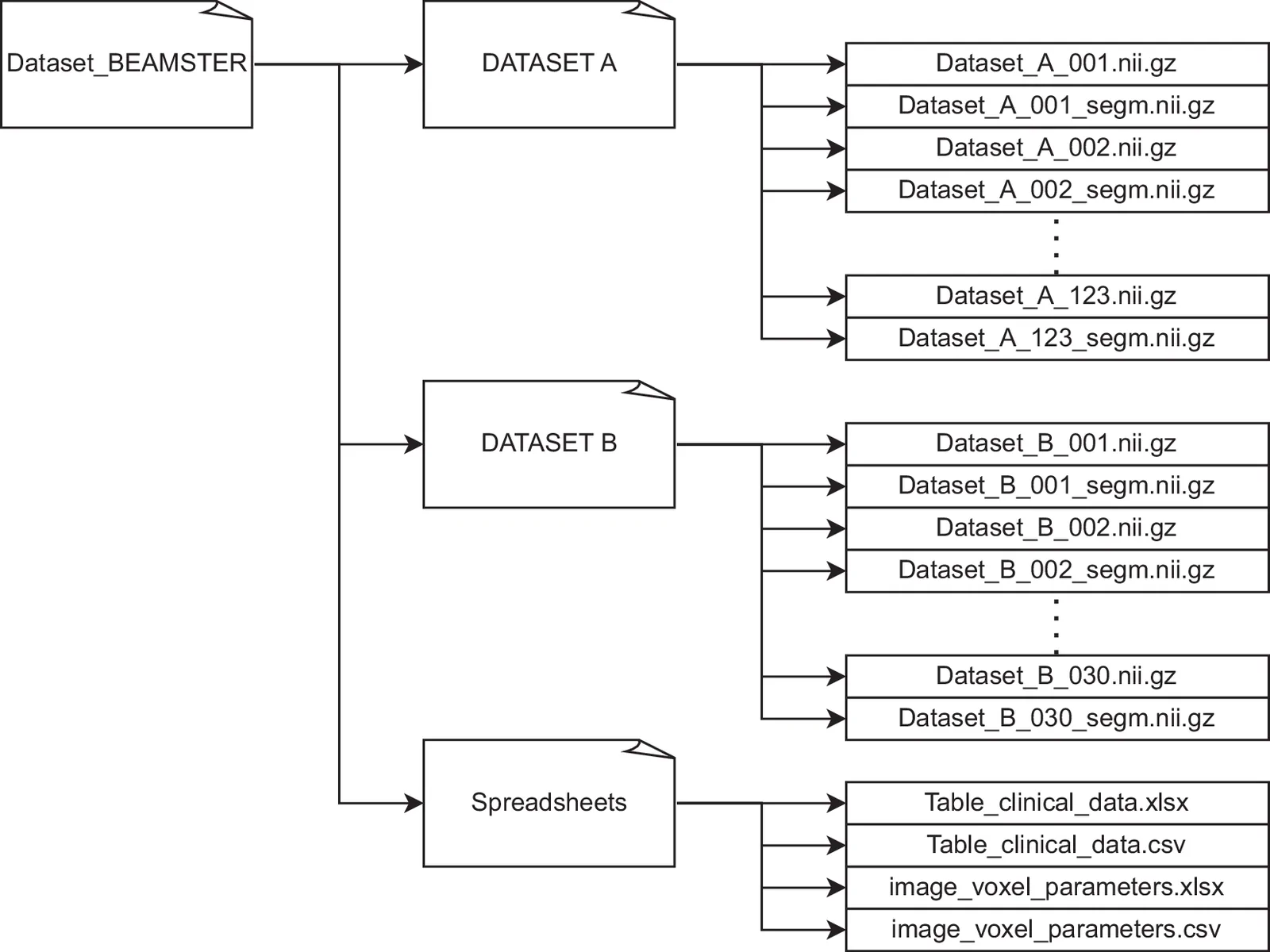
Dataset structure. The resource, referred to as Dataset_BEAMSTER, is organized into two imaging subsets (Dataset A and Dataset B), each containing patient-wise contrast-enhanced T1-weighted MRI scans in NIfTI format (.nii.gz) and the corresponding binary segmentation masks (.nii.gz). In addition, a Spreadsheets folder provides curated clinical data (CSV/XLSX) and voxel-level acquisition parameters. This organization ensures a clear link between imaging data, expert annotations, and associated clinical information.

**Fig. 3:**
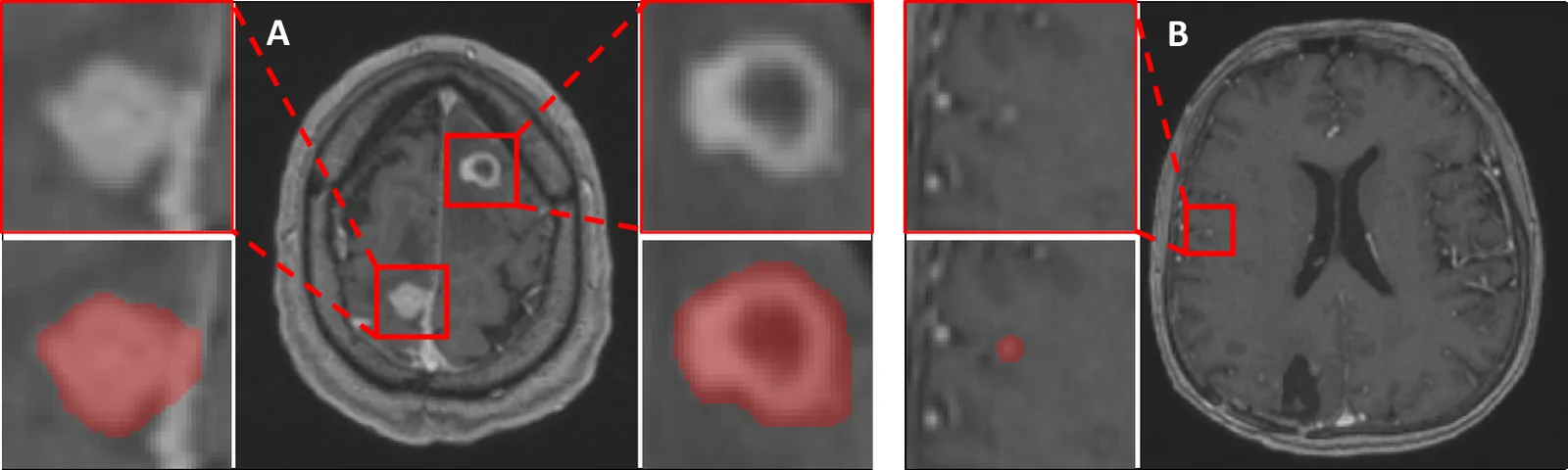
Example of available MRI data and corresponding expert segmentations. A (left) shows a case from Dataset A with two contrast-enhancing brain metastases; zoomed regions highlight the original T1-weighted MRI and the corresponding binary segmentation masks. B (right) illustrates a case from Dataset B containing a small metastatic lesion, with the zoomed view demonstrating the original image and its segmentation overlay.

An overview of the dataset structure is provided in Fig. 2, while Fig. 3 shows an example of the imaging data and corresponding expert annotation.

### Data overview

The dataset comprises 140 brain MRI examinations of patients with brain metastases, divided into Dataset A (113 cases) and Dataset B (27 cases enriched with small lesions to increase segmentation difficulty).

Each case includes a contrast-enhanced T1-weighted MRI scan and a corresponding expert-annotated binary segmentation mask. In addition, structured clinical metadata are provided for each subject.

## Technical Validation

The creation of this dataset involved several validation steps to ensure image quality, correct anonymization, and accurate annotation of the lesion. The image quality was reviewed during the export process and only diagnostically usable MRI scans were included. The successful de-identification, conversion from DICOM to NIfTI, and anonymization by defacing with AFNI software were independently validated by a clinical engineer from the Department of Oncology and by an external engineering specialist.

The segmentation precision was verified by a board-certified radiologist with extensive clinical experience in neuro-radiology. Annotations were performed in a clinical radiotherapy planning system under routine conditions, following international guidelines for the definition of the target volume. For this study, the exported structures were checked for consistency and correctness after conversion to binary NIfTI masks. Clinical metadata (age, sex, diagnosis, treatment information) were manually curated and cross-checked for consistency.

Despite these validation steps, some limitations remain. An inherent challenge is the reproducibility of the annotation of the lesion, particularly for small or ambiguous metastases that may be difficult to delineate even for experienced neuroradiologists. The variability between observers was not formally assessed in this dataset, and the annotations represent the judgment of a single expert. However, the inclusion of small lesions increases the clinical relevance of the dataset and provides a valuable benchmark for the development of robust detection and segmentation algorithms.

## Usage Notes

This dataset provides T1-weighted MRI scans of brain metastases together with expert binary segmentation masks. It is particularly suitable for training, validation, and benchmarking of automated algorithms for brain metastasis detection and segmentation. An important feature of the collection is the inclusion of small metastatic lesions, which represent a common challenge for both radiologists and machine learning models. This makes the dataset valuable for developing methods that are sensitive not only to large, well-defined lesions but also to subtle, small-volume metastases.

Data are provided in NIfTI format, which is widely supported by medical imaging software such as ITK-SNAP^21^, MITK^22^, or other appropriate tools. The binary segmentation masks can be directly used as ground-truth labels for supervised machine learning pipelines. Researchers may also integrate the dataset with other publicly available resources, such as BraTS-METS^10^, BrainMetShare^12,13^, or UCSF-BMSR^15^, to perform comparative studies or to improve the generalizability of the model.

All imaging data have been fully anonymized and both MRI and segmentation masks are organized in a subject-wise structure to facilitate straightforward reuse. The dataset, therefore, provides a flexible resource for computer vision, radiomics, and clinical research communities aiming to advance methods for brain metastasis analysis.

## Data Availability

The complete dataset is available in the (https://doi.org/10.6084/m9.figshare.29365844) FigShare repository and is licensed under CC-BY 4.0.

## Code availability

The conversion of the imaging data from DICOM to NIfTI format was performed using the SimpleITK Python library^23^. Segmentation masks were extracted from DICOM RT structure sets with the open-source DicomRTTool package^24^.
